# Defining indicators for the scoping stage of health impact assessment to evaluate tobacco control policy in the city of Beijing

**DOI:** 10.1186/s12889-023-15982-4

**Published:** 2023-06-06

**Authors:** Yunting Zheng, Yuhui Shi, Ying Ji, Xiurong Liu, Haoxiang Lin, Meijun Chen, Qingping Yun, Chun Chang

**Affiliations:** 1 School of Health Management, Fujian Medical University, Fuzhou, 350122 China; 2 Department of Social Medicine and Health Education, School of Public Health, Peking University, No. 38 Xueyuan Road, Beijing, 100191 China; 3 Beijing Center for Disease Prevention and Control, Beijing, 100013 China; 4 University of Hong Kong, Hongkong, 999077 China; 5 Shenzhen Institutes of Advanced Technology, Shenzhen, 518055 China

**Keywords:** Health Impact Assessment, Tobacco Control, Indicator Set

## Abstract

**Introduction:**

Beijing initiated the nation’s most comprehensive tobacco control program that adheres to the WHO Framework Convention on Tobacco Control. This study aimed to identify a set of indicators for the scoping of an Health Impact Assessment (HIA) to assess this policy.

**Methods:**

This study used a modified Delphi process. It proposed a tobacco control health impact framework based on the Driving forces- Pressure- State- Exposure- Effect- Action model and the Determinants of Health Theory. After a review of current surveillance system and literature, a working group of 13 experts with multidisciplinary background was established to formulate indicator evaluation criteria and conduct indicator scoring. Each indicator was scored by experts according to four evaluation criteria chosen. Indicators that obtained a total score above 80% and with standard error less than 5 were selected as the final set of indicators. Kendall’s coefficient of concordance was calculated.

**Results:**

Twenty-three out of 36 indicators were selected. Smoking prevalence, mortality rate, hospital admission rate, tobacco consumption and hospital admission fees of smoking related diseases achieved more than 90% of total scores and ranked as the top five. Kendall’s concordance coefficient was 0.218 for all indicators. For all model composition, Kendall’s concordance coefficients were statistically significant.

**Conclusion:**

This study identified a set of twenty-three indicators for scoping of HIA of a comprehensive tobacco control policy in Beijing based on a tobacco control health impact conceptual framework. The set of indicators achieved high scores and statistically significant consistency and has great potential to promote the evaluation of tobacco control policy in a global city. Further study might use the set of indicators for HIA on tobacco control policy to analyze empirical data.

## Introduction

China is the world’s biggest tobacco consumer, with an estimated consumption that exceeds that of the 39 countries combined [1]. In 2018, there were more than 300 million smokers in China, with a smoking prevalence of 50.5% for men and 2.1% for women [2]. According to estimates, 68.1% of the surveyed of nonsmokers in China experience SHS at least once each day [2]. In China, smoking contributed to roughly 2 million fatalities in 2017 [3]. China signed the Framework Convention on Tobacco Control (FCTC) of the World Health Organization (WHO) in 2005, and it went into effect in 2006. However, it has taken a little while to fulfill the responsibilities [4]. Due to China’s tobacco monopoly, the FCTC has made only modest national progress [5], and there is no smoking ban in public places [5, 6].

As seen by the 21 cities in China that have passed smoke-free regulations in recent years, there is a rising bottom-up approach to tobacco control legislation in that country [7], including Beijing. For the smoke-free Olympics, Beijing implemented a smoking ban in 11 different types of public spaces in 2008 [8]. Beijing residents’ smoking rate has fallen by 1.5% as a result of the regulation [9] however, the smoking phenomenon quickly returned to its previous status after the Olympics [10]. Beijing implemented the *Beijing Municipal Tobacco Control Regulation* in June 2015, seven years later [11]. The National Tobacco Tax Reform was started in May 2015 almost simultaneously [12], which, by the middle of 2015, has created the Beijing comprehensive tobacco control policy. Six MPOWER measures, including *Monitoring tobacco use, Protect people from tobacco smoking, Offer help to quit tobacco, Warning about the dangers of tobacco, Enforcing tobacco advertising, promotion & sponsorship, Raising taxes on tobacco*, as recommended by WHO, make up Beijing’s comprehensive tobacco control policy. These include a ban on smoking in all indoor and four outdoor public places, a higher tobacco tax, a ban on tobacco advertising, promotion, and sponsorship, the establishment of a cessation support system, and a media campaign [13]. In 2015, this was the nation’s most successful tobacco control strategy that followed the WHO standard.[14]. The introduction of the 2015 policy have decreased the amount of cigarettes sold per person between 2015 and 2017 by 1388.2 sticks [15]. From 2014 to 2017, there was a further decline in smoking prevalence and SHS exposure, with decreases of 20.3% and 25.6%, respectively [16]. After the adoption of smoke control measures, 18,137 (26.7%) hospital admissions for stroke decreased [13] and 5581 (17.5%) hospital admissions for chronic obstructive pulmonary disorders [17] were probably avoided in Beijing for 25 months.

Health Impact Assessment (HIA) is a combination of procedures, methods and tools by which a policy, program or project may be judged as to its potential effects on the health of a population, and the distribution of those effects within the population [18]. HIA has been used extensively to assess the health impacts of major national policies and inform the policy making process [19]. Basic steps for carrying out an HIA include screening, scoping, appraisal, reporting, and monitoring. Screening means making a quick mapping of whether there are potential linkages between the policy, program or project and health, and what different aspects of health they might affect. Scoping is intended to identify how the HIA will be carried out and to set the boundaries for the assessment. Appraisal means rapid or in-depth assessment of health impacts using available evidence – who will be affected, what is the baseline, what is the prediction, significance and mitigation. Reporting contains conclusions and recommendations to remove or mitigate negative impacts on health or to enhance positive impacts. Monitoring is where appropriate, to monitor actual impacts on health to enhance existing evidence. The existing scoping approach lace a clear, systematic method of selection of indicators in HIA [20].

Costa et al. applied the HIA methodology on the Portuguese law on Smoking Prevention and Tobacco Control including indicators: rates of total and premature tobacco associated mortality, standardized mortality rate for all tobacco related diseases, the number of hospital admissions for ischaemic heart disease and cerebrovascular disease, the number of patients diagnosed with chronic obstructive pulmonary disease (COPD), smoking prevalence and the total number of smoking cessation consultations [21]. Though it measured the change of some indicators after a tobacco control policy, the selection procedure of indicators was not mentioned and whether these indicators were comprehensive remained unknown. The WHO recommended four essential indicators [22] for measuring the effect of the tobacco control legislation on “outcomes”: mortality, tobacco consumption, smoking prevalence and tobacco control policies. But it focused on outcome evaluation without including process evaluation and only four indicators were recommended. While Beijing’s tobacco control policy employed many local measures, such as tobacco control complaint, which generated a series of process indicators, so we feel there was a need for a comprehensive set of indicators to measure the impact of tobacco control policy in Beijing based on a scientific selection procedure.

This study aimed to identify a set of indicators for the scoping of an HIA to assess Beijing’s comprehensive tobacco control policy and serve as a reference for future HIAs on tobacco control policy in China and perhaps in other developing countries. Results of HIA would be summarized in further publication.

## Methods

**Fig. 1:**
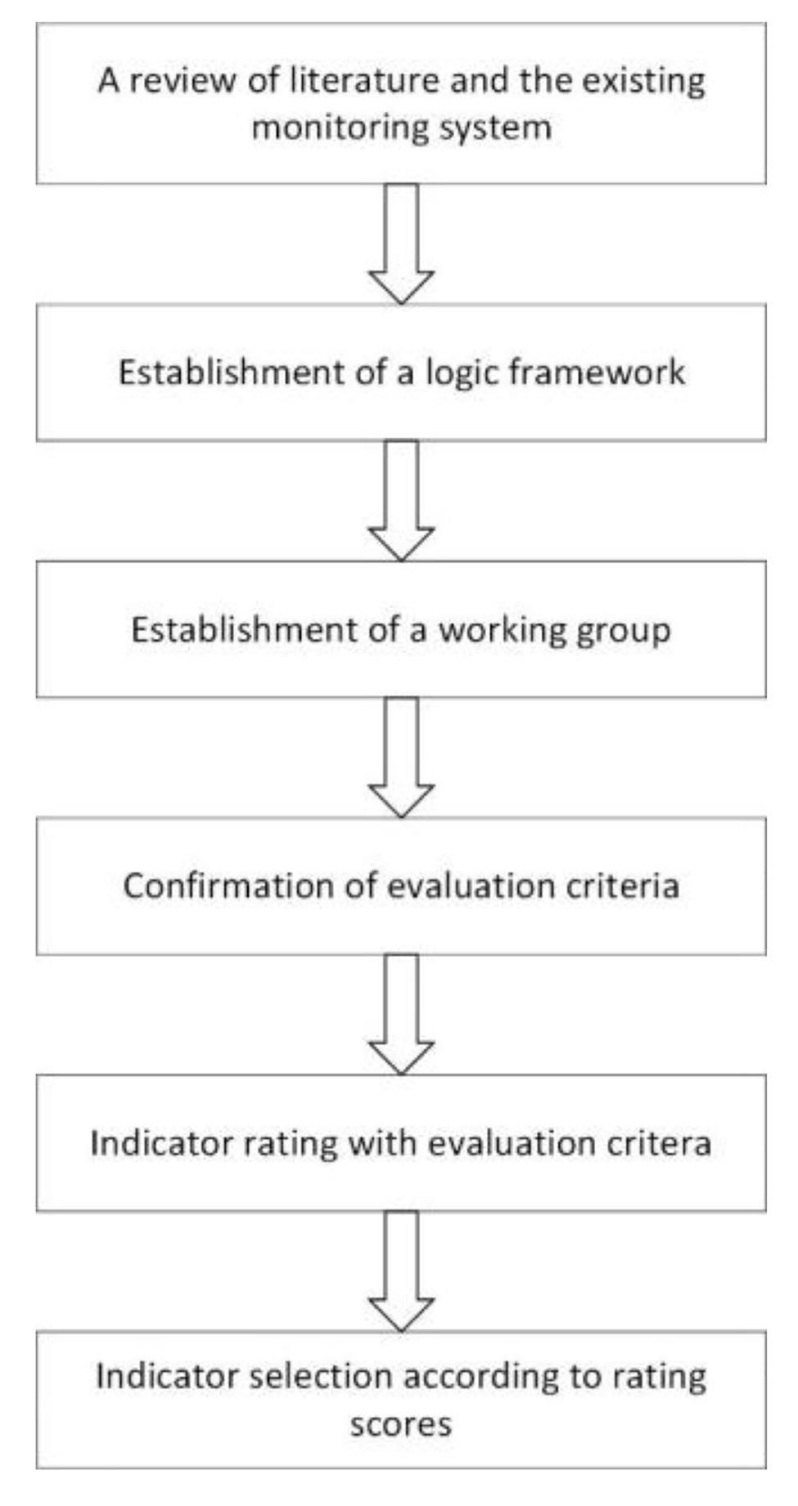
Flow chart of the study process

This study used a modified Delphi process. Our research process was illustrated in the flow chart in Fig. 1. Firstly, a review was performed of the existing monitoring system of tobacco control in Beijing and of indicators identified from the literature by our research team. We systematically searched the following databases for studies: Medline, The Cochrane Library and Web of Science. Because of the large volume of potentially relevant research in China, we also searched Chinese databases: China National Knowledge Infrastructure Database and Wanfang Database. The current study used the following keywords for searching: health impact assessment, smoke-free, smoking ban, anti-smoking, *Jian Kang Ying Xiang Ping Jia, Wu Yan Zheng Ce, Kong Yan, Bei Jing Shi Kong Yan Tiao Li*. This study used a modified PICOT (Population, Intervention, Comparison, Outcome, and Time) format [23] to summarize the inclusion criteria of literature review by replacing the Comparison with the Setting. The inclusion criteria of literature review were described as below:

Population: residents in certain region.

Intervention: smoking-free law/smoking ban/tobacco control policy/MPOWER measures.

Setting: city/state/province/country within member states that signed the FCTC.

Outcome: process or outcome evaluation indicators.

Time period: 2003–2020.

Exclusion criteria included:

Population: migrant population.

Intervention: non-MPOWER measures.

Setting: non-member states of the WHO or states that did not signed the FCTC.

Outcome: non-evaluation indicators.

Time period: out of the timeframe 2003–2020.

**Fig. 2:**
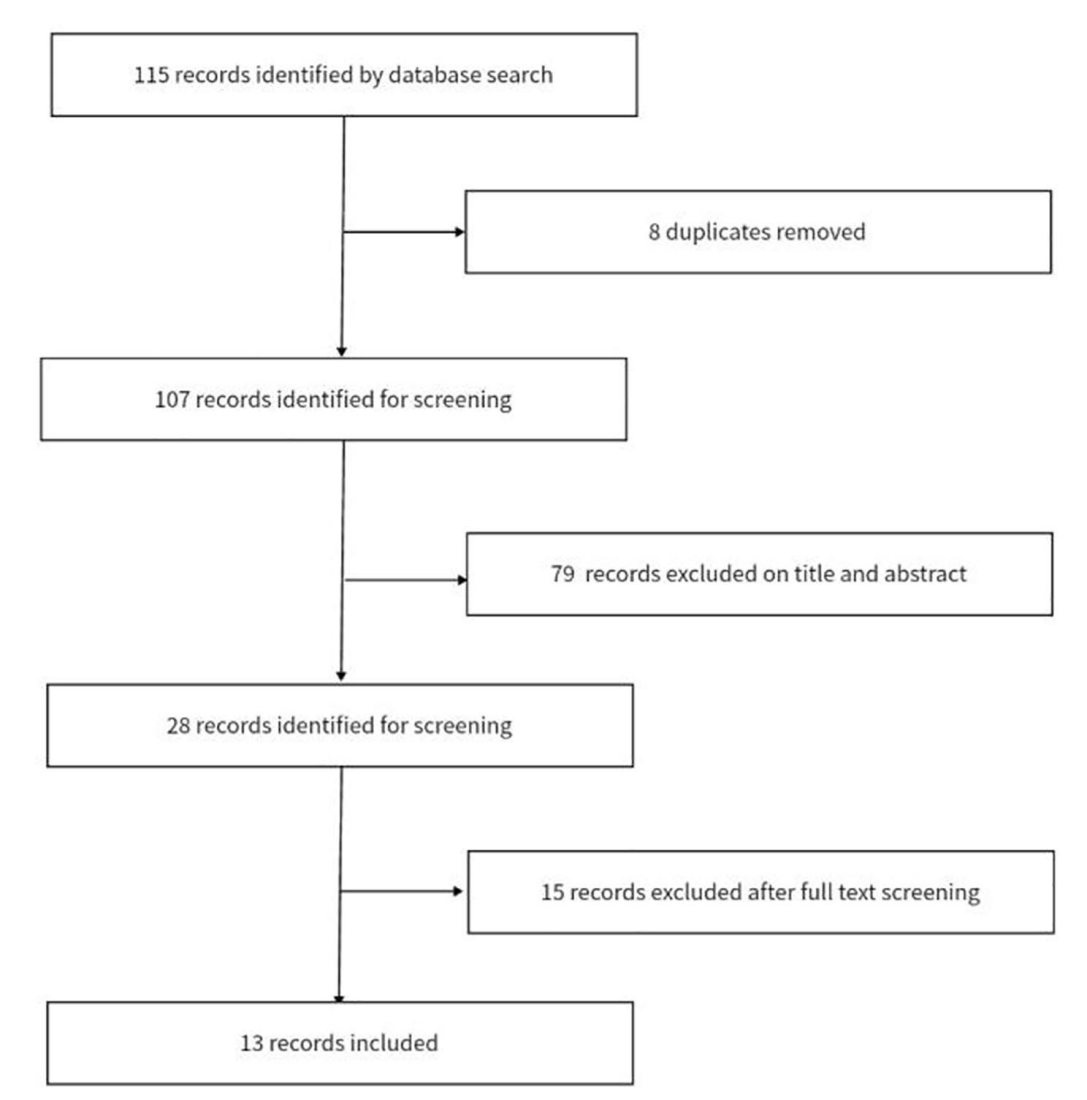
Flow chart of the literature screening process

The last date for literature search was September 20, 2020. The literature found was reviewed by two independent researchers to decide whether a study met the inclusion criteria, and discrepancies were discussed until agreements were reached. Procedure of literature screening was presented in Fig. 2. For studies included for the review, indicators including both process and outcome indicators were collected by one researcher.

**Fig. 3:**
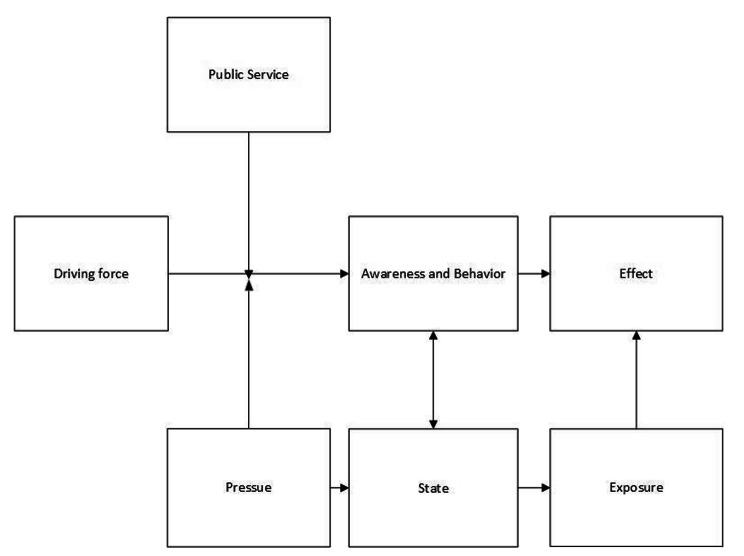
Conceptual framework for tobacco control Health Impact Assessment

Secondly, the study developed a tobacco control policy HIA framework (Fig. 3) based on the DPSEEA (Driving forces, Pressure, State, Exposure, Effect, Action) model [24] and the Determinants of Health (DOH) model [25]. The DPSEEA framework was used for the development of environmental health indicators. One of the main purposes of tobacco control policy is to reduce second hand-smoke which is an environmental health issue, so the DPSEEA framework is suitable for tobacco control policy. The DOH complemented the DPSEEA framework by providing dimensions on actions by individuals (e.g. active smoking and quitting) and on public services (e.g. tobacco control publicity and cessation services). Combining the two models could capture the most information of Beijing’s comprehensive tobacco control policy. The DPSEEA framework and the DOH have been used as a way of selecting and to structure environmental and human health indicators, respectively. The DPSEEA framework contains six components: (a) driving force: factors which motivate and push the environmental processes involved, (b) pressure: pressures on the environment generated as result of the driving forces, (c) state: environmental levels modified in response to the pressures, (d) exposure: human exposure to environmental hazards, (e) effect: health effects led by the exposure to environmental hazards and (f) action: society attempts to invoke a range of actions in the face of these effects. [24]. The DOH [25] illustrates the main influences on health, including (a) major structural environment: include economic strategies, tax policies, trade and environmental agreements between countries, (b) material and social conditions in which people live and work, determined by various determinants such as housing, education and public services like health care, (c) mutual support from family, friends, neighbors and the local community, (d) actions taken by individuals, such as smoking habits, (e) age, sex and genetic make-up of each individual. With the goal of controlling tobacco consumption, strategies could involve four levels: (a) cigarette taxation, (b) bans on cigarette advertising and the creation of smoke-free public places, (c) helping communities join together to press for tighter controls on sales of cigarette to children in local shops, (d) the education of the general public about the dangers of smoking.

The proposed tobacco control health impact framework consists of driving force, public services, pressures, awareness and behaviors, states, exposures and effects (Fig. 2). It recognizes that the link between driving force – the tobacco control policy and health effects is determined by many different factors operating through a chain. Driving force refers to factors that promote the effective implementation of tobacco control policy. Public service refers to the provision of public services due to policy changes. Pressure comes at all stages of the tobacco supply chain. Awareness and behavior refer to people’s awareness level and active behavior. Status refers to the state of environmental hazards. Exposure refers to the interaction between people and environmental hazards. Effect refers to health effects arising from behavior or exposure to environmental hazards. Based on the developed conceptual framework, the study integrated the indicators identified through the review.

Thirdly, a working group was established representing tobacco control operators and decision makers, HIA experts, statistician and data providers. The working group formulated the evaluation criteria of the indicators and conducted scoring of the indicators. The initial evaluation criteria were taken from Sara Farchi’s [26] research on defining a common set of indicators to monitor road accidents in the European Union. The working group discussed the evaluation criteria, made necessary adjustments according to China’s context and the tobacco control topic and finally approved the evaluation criteria as below: (a) a clear and commonly accepted definition (the degree of clearness and acceptancy by the public); (b) importance (degree of importance to realize research objectives); (c) accuracy (degree to which measured values reflect to actual values); (d) stability (degree to which it is not affected by other factors).

Lastly, the indicators proposed were rated with the evaluation criteria independently by each expert of the working group, the possible values ranged from 0 to 5. For results analysis, the mean and standard deviation of the rate was calculated. A score was calculated as the sum of the mean scores obtained by each criterion, which had a potential range of 0 to 20. Indicators that obtained a total score above 16 and with standard error less than 5 were selected as the final set of indicators. The threshold of 16 score (80% of total score) is stricter than Farchi’s study (62.5%) [26] and we also consider standard error threshold of 25% (a quarter) for consistency between experts. Kendall’s coefficient of concordance was calculated by IBM SPSS (version 24).

## Results

### Review of existing monitoring system and literature

The monitoring system reported 32 indicators from all dimensions of our proposed frameworks (Fig. 2), including: (1) driving force – (a) number of complaints through complaint hotline and social media complaint platform, (b) times of enforcement supervision of tobacco control, (c) posting rate of no-smoking signs in public places; (2) public service – (a) number of smoking cessation clinics, (b) percentage of residents who ever have seen or listen to tobacco control information through media, (c) person-time of receiving cessation intervention in medical institutions; (3) pressure – (a) cigarette consumption, (b) cigarette price, (c) cigarette price elastic coefficient, (d) cigarette tax; (4) awareness and behavior – (a) smoking prevalence, (b) awareness rate of smoking hazard, (c) cessation rate, (d) planning cessation rate; (4) status - “Three-without” indicator; (5) exposure – (a) second-hand smoke exposure, (b) content of cotinine in non-smokers’ saliva; (6) effect – (a) incidence of smoking related diseases, (b) premature death risk of smoking related diseases.

Nine indicators were identified from the literature reflecting four dimensions of our framework: pressure, awareness and behavior, state, exposure and effect dimensions. Indicators included cigarette consumption[27, 28]; smoking prevalence [29, 30]; second-hand exposure [31]; indoor air quality [32]; hospital admission rate and hospital admission fee of cardiovascular diseases [33, 34]; hospital admission rate and hospital admission fee of respiratory diseases [35, 36]; mortality for cardiovascular diseases [37, 38] and lung cancer [39]. In total, 36 indicators were found through the review including 5 indicators were identified through both monitoring system and literature.

### Basic characteristics of experts of working group

Basic characteristics of experts of working group was summarized in Table 1. Experts in this study presented a high level of authority. Over 70% of them ranked associate professor or professor, more than 60% of which held a master or higher degree, over 70% of them held a title of deputy director or director, and 61.6% had over 21 years of working experience. Experts enjoyed a multidisciplinary background, with expertise in tobacco control, HIA, health policy research and statistics. Around one third of experts were from government agencies, one third were from Non-Governmental Organizations (NGOs) and the others were from academic institutions.

**Table 1:** Background characteristics of experts (N = 13)

Item	N	(%)
**Socio-demographics**		
Gender		
Male	5	38.5
Female	8	61.5
Age (years old)		
36–45	4	30.8
> 45	9	69.2
Highest education obtained		
PhD	1	7.7
Master	7	53.8
Bachelor	5	38.5
Major		
Epidemiology and health statistics	3	23.1
Social medicine and health administration	3	23.1
Health education and health promotion	3	23.1
Public health	2	15.4
Global health	1	7.7
Clinical medicine	1	7.7
**Academic background**		
Professional title		
Professor	2	15.4
Associate professor	8	61.5
Lecturer	3	23.1
Job title		
Director	3	23.1
Deputy director	7	53.8
Others	3	23.1
Working area		
Tobacco control	3	23.1
Health impact assessment	7	53.8
Health policy research	1	7.7
Statistics	2	15.4
Working experience		
< 10	1	7.7
11–20	4	30.8
21–30	4	30.8
> 30	4	30.8
Eligibility as supervisor		
PhD supervisor	3	23.1
Master supervisor	6	46.2
None of the above	4	30.8

### Evaluation results

To select the final set of indicators, all the hypothetical indicators were screened according to the criteria defined (Table 2). Table 3 presents the results of indicator evaluation. Smoking prevalence (19.6 ± 0.8), mortality rate (19.5 ± 1.2), hospital admission rate (18.9 ± 2.2), tobacco consumption (18.5 ± 2.4) and hospital admission fees of smoking related diseases (18.4 ± 2.7) achieved more than 90% of total scores and ranked as the top five while the number of complaints through social media complaint platform (14.2 ± 4.9) ranked last. Smoking prevalence ranked first among three evaluation criteria: definition clearness and commonly acceptance (5.0 ± 0.0), importance (5.0 ± 0.0) and accuracy (4.9 ± 0.4) and mortality rate (4.8 ± 0.4) ranked first in stability. The person-time of audiences of tobacco control publicity achieved the lowest scores among all four criteria.

**Table 2:** Definition and measurement of hypothetical indicators

Model position	Indicator	Definition	Measurement
Driving force	Number of complaints through complaint hotline	Tobacco control complaints person-time through Beijing’s public health hotline - “12,320”	The number of calls to 12,320 on tobacco control complaints in Beijing
	Number of complaints through social media complaint platform	Tobacco control complaints person-time through Wechat complaint platform - “Smoke-free Beijing”	The number of complaints submissions through “Smoke-free Beijing”
	Times of enforcement supervision of tobacco control	Times of enforcement supervision by Beijing’s health supervision system	The number of tobacco control enforcement supervision completed by Beijing’s health supervision system
	Posting rate of no-smoking signs in public places	Percentage of public places positing no-smoking signs	Number of public places positing no-smoking signs/Number of public places surveyed in Beijing
Public service	Percentage of residents who ever have seen or listen to tobacco control information through media	Percentage of Beijing’s adults having ever seen or listen to tobacco control information through media	Number of residents having ever seen or listen to tobacco control information/Number of residents surveyed in Beijing
	Number of tobacco control publicity materials distributed	Number of all kinds of tobacco control publicity materials distributed in Beijing	Number of tobacco control publicity materials distributed by tobacco control sector in Beijing
	Rate of doctors advise smoking cessation	Percentage of Beijing’s current smokers’ who received doctors advise on smoking cessation in the last 12 months	Number of current smokers’ who received doctors advise on cessation/Number of current smokers surveyed in Beijing
	Number of smoking cessation clinics	Number of smoking cessation clinics in Beijing	Number of smoking cessation clinics with record in Beijing’s health system
	Person-time of receiving cessation intervention in medical institutions	Person-time of receiving cessation intervention in medical institutions in Beijing	Person-time of receiving cessation intervention in medical institutions with record in Beijing’s health system
Model position	Indicator	Definition	Measurement
Public service	Person-time of brief cessation intervention	Person-time of brief cessation intervention in Beijing	Person-time of receiving brief cessation intervention in medical institutions recorded in Beijing’s health system
	Person-time of cessation services by smoking cessation clinics	Person-time of cessation services by smoking cessation clinics in Beijing	Person-time of receiving cessation services in cessation clinics recorded in Beijing’s health system
	Person-time of services through smoking cessation hotline	Person-time of services through smoking cessation hotline in Beijing	Person-time of cessation cervices through 12,320 cessation hotline in Beijing
Pressure	Percentage of residents who see tobacco advertisements	Percentage of Beijing’s adults who ever see tobacco advertisements	Number of residents who ever see tobacco advertisements/Number of residents surveyed in Beijing
	Percentage of residents who see tobacco promotion	Percentage of Beijing’s adults who ever see tobacco promotion	Number of residents who ever see tobacco promotion/Number of residents surveyed in Beijing
	Cigarette price	Cigarette price in Beijing	Retail price of cigarette in Beijing
	Cigarette tax	Cigarette tax in Beijing	Tax revenue of cigarette in Beijing
	Cigarette consumption	Beijing’s cigarette consumption per capita	Sales volume of cigarette in Beijing
	Cigarette affordability	Cost of buying 100 packs of cigarettes as a percentage of revenue in Beijing	Cost of buying 100 packs of cigarettes/GDP per capita in Beijing
	Cigarette price elastic coefficient	Change in percentage of cigarette sales as a result of a change in price	Change in percentage of cigarette sales/change in cigarette price
Awareness and behavior	Awareness rate of the hazards of smoking among residents	Beijing’s adults’ awareness rate of smoking hazards	Number of residents correctly answer the hazards of smoking/Number of residents surveyed in Beijing
	Awareness rate of tobacco control policy	Beijing’s adults’ awareness rate of tobacco control policy in public places	Number of residents who are aware of tobacco control policy in public places/Number of residents surveyed in Beijing
Model position	Indicator	Definition	Measurement
Awareness and behavior	Supporting rate of tobacco control policy	Beijing’s adults’ supporting rate of tobacco control policy in public places	Number of residents support tobacco control policy in public places/Number of residents surveyed in Beijing
	Smoking prevalence	Current smoking prevalence among Beijing’s adults	Number of current smokers/Number of residents surveyed in Beijing
	Cessation rate	Cessation rate among Beijing’s ever smokers	Number of ever smokers who successfully quit/Number of ever smokers surveyed in Beijing
	Planning cessation rate	Percentage of current smokers who consider quitting in the next 12 months	Number of current smokers who consider quitting in the next 12 months/Number of current smokers in Beijing
	Attempting cessation rate	Percentage of current smokers who have ever tried to quit at least once in the past 12 months	Number of current smokers who have ever tried to quit at least once in the past 12 months/Number of current smokers surveyed in Beijing
Status	Indoor pollutant level	Indoor pollutant levels at sampling sites	Content of pollutant in the air at sampling sites
	“Three without” indicator	Percentage of public places without smell of cigarette, butt or smoking set	Number of public places without smell of cigarette, butt or smoking set/Number of public places surveyed
Exposure	Second-hand smoke exposure rate	Secondhand smoke exposure rate among adults in public places	Number of residents ever exposed to secondhand smoke/Number of residents who do not smoke
	Active dissuasion rate	Percentage of adults in Beijing who actively discourages smokers from smoking when seeing them	Number of residents who actively discourage smokers from smoking when seeing them/Number of residents surveyed in Beijing
	Content of cotinine in non-smokers’ saliva	Cotinine levels in saliva among non-smoking restaurant staff	Content of cotinine in saliva among non-smoking restaurant staff surveyed
Model position	Indicator	Definition	Measurement
Effect	Hospital admission rate of smoking related diseases	Hospital admission rate of smoking related diseases among Beijing’s adults	Number of hospital admission of smoking related diseases/Number of adult population in Beijing
	Hospital admission fees of smoking related diseases	Hospital admission fees of smoking related diseases among Beijing’s adults	Hospital admission fees of smoking related diseases in Beijing
	Mortality rate of smoking related diseases	Mortality rate of smoking related diseases among Beijing’s adults	Number of death of smoking related diseases/Number of adult population in Beijing
	Incidence of smoking related diseases	Incidence of smoking related diseases among Beijing’s adults	Number of new cases of smoking related diseases/Number of adult population in Beijing
	Premature death risk of smoking related diseases	Premature death risk of smoking related diseases among Beijing’s adults	1-∏^69^_age 30_(1-Probability of death in a certain age group)

**Table 3:** Results of indicator evaluation

Model position	Indicator	Evaluation criteria (Mean ± SD)				
		A clear and commonly accepted definition	Importance	Accuracy	Stability	Overall
Driving force	Number of complaints through complaint hotline	4.5 ± 1.1	4.2 ± 1.2	3.9 ± 1.1	3.8 ± 1.0	16.3 ± 4.0
	Number of complaints through social media complaint platform	3.8 ± 1.5	3.6 ± 1.3	3.5 ± 1.3	3.3 ± 1.2	14.2 ± 4.9
	Times of enforcement supervision of tobacco control	4.7 ± 1.1	4.4 ± 1.2	4.2 ± 1.2	4 ± 1.2	17.3 ± 4.4
	Posting rate of no-smoking signs in public places	4.3 ± 0.9	4.3 ± 0.9	4.4 ± 0.8	4.1 ± 1.0	17.1 ± 3.1
Public service	Percentage of residents who ever have seen or listen to tobacco control information through media	4.3 ± 0.6	4.0 ± 0.8	3.9 ± 0.9	3.9 ± 0.9	16.2 ± 2.7
	Number of tobacco control publicity materials distributed	4.2 ± 0.8	3.5 ± 0.8	3.6 ± 0.8	3.4 ± 0.9	14.6 ± 2.7
	Rate of doctors advise smoking cessation	4.2 ± 0.9	4.2 ± 0.8	3.6 ± 0.8	3.7 ± 0.8	15.6 ± 2.7
	Number of smoking cessation clinics	4.5 ± 0.7	4.2 ± 0.8	4.3 ± 0.8	4.2 ± 0.8	17.2 ± 2.7
	Person-time of receiving cessation intervention in medical institutions	4.4 ± 0.5	4.2 ± 0.7	3.5 ± 0.5	3.5 ± 0.5	15.5 ± 1.2
	Person-time of brief cessation intervention	4.2 ± 0.8	4.1 ± 0.9	3.2 ± 0.8	3.1 ± 0.8	14.5 ± 2.9
Public service	Person-time of cessation services by smoking cessation clinics	4.5 ± 0.7	4.3 ± 0.8	3.9 ± 1.0	3.6 ± 1.2	16.4 ± 3.1
	Person-time of services through smoking cessation hotline	4.4 ± 1.0	3.9 ± 1.0	3.7 ± 1.0	3.7 ± 1.0	15.7 ± 3.6
Pressure	Percentage of residents who see tobacco advertisements	4.3 ± 1.0	4.0 ± 0.9	3.5 ± 1.0	3.5 ± 0.9	15.4 ± 3.3
	Percentage of residents who see tobacco promotion	4.1 ± 1.0	4.0 ± 0.9	3.4 ± 0.9	3.4 ± 0.8	14.8 ± 3.1
	Cigarette price	4.4 ± 0.8	4.5 ± 0.7	4.2 ± 1.1	4.2 ± 1.1	17.2 ± 3.0
	Cigarette tax	4.5 ± 0.8	4.2 ± 0.9	4.0 ± 1.1	3.9 ± 1.3	16.5 ± 3.8
	Cigarette consumption	4.7 ± 0.6	4.7 ± 0.6	4.6 ± 0.7	4.5 ± 0.7	18.5 ± 2.4
	Cigarette affordability	4.3 ± 0.8	4.0 ± 0.7	3.6 ± 0.8	3.7 ± 0.8	15.6 ± 2.5
	Cigarette price elastic coefficient	4.5 ± 0.8	4.5 ± 0.8	3.8 ± 0.8	3.8 ± 0.7	16.6 ± 2.6
Awareness and behavior	Awareness rate of the hazards of smoking among residents	4.7 ± 0.6	4.7 ± 0.6	4.2 ± 0.7	4.3 ± 0.8	17.9 ± 2.3
	Awareness rate of tobacco control policy	4.4 ± 0.8	4.7 ± 0.6	4.2 ± 0.9	4.2 ± 0.8	17.5 ± 2.4
	Supporting rate of tobacco control policy	4.2 ± 0.9	4.4 ± 0.9	4.1 ± 1.0	4.2 ± 0.9	16.8 ± 3.2
Status	Smoking prevalence	5.0 ± 0.0	5.0 ± 0.0	4.8 ± 0.4	4.8 ± 0.4	19.6 ± 0.8
	Cessation rate	4.5 ± 0.7	4.5 ± 0.7	4.1 ± 1.0	4 ± 1.0	17.0 ± 3.3
Status	Planning cessation rate	4.5 ± 0.8	4.2 ± 0.9	3.7 ± 1.0	3.6 ± 1.0	16.0 ± 3.2
	Attempting cessation rate	4.2 ± 0.9	3.8 ± 1.1	3.6 ± 1.0	3.6 ± 1.0	15.2 ± 4.0
	Indoor pollutant level	4.3 ± 0.9	4.4 ± 1.0	4 ± 1.0	4 ± 1.0	16.7 ± 3.6
	“Three without” indicator	4.5 ± 0.9	4.3 ± 0.9	3.5 ± 1.1	3.7 ± 0.9	16.0 ± 3.3
Exposure	Second-hand smoke exposure rate	4.6 ± 0.8	4.6 ± 0.8	4.0 ± 0.8	3.9 ± 1.0	17.2 ± 2.5
	Active dissuasion rate	4.3 ± 0.9	4.2 ± 0.9	3.5 ± 0.8	3.4 ± 0.8	15.3 ± 2.9
Effect	Content of cotinine in non-smokers’ saliva	4.3 ± 1.0	4.0 ± 1.0	4.0 ± 1.3	4.1 ± 1.0	16.4 ± 3.9
	Hospital admission rate of smoking related diseases	4.8 ± 0.6	4.8 ± 0.6	4.6 ± 0.7	4.5 ± 0.7	18.8 ± 2.2
Effect	Hospital admission fees of smoking related diseases	4.8 ± 0.6	4.7 ± 0.6	4.5 ± 0.9	4.4 ± 0.9	18.4 ± 2.7
	Mortality rate of smoking related diseases	4.9 ± 0.3	4.9 ± 0.3	4.8 ± 0.4	4.8 ± 0.4	19.5 ± 1.2
	Incidence of smoking related diseases	4.4 ± 0.9	4.4 ± 0.8	3.8 ± 0.6	4.1 ± 0.9	16.6 ± 2.3
	Premature death risk of smoking related diseases	4.2 ± 0.9	4.4 ± 0.7	3.7 ± 0.6	3.8 ± 0.8	16.1 ± 2.1

Table 4 presents twenty-three indicators with evaluation scores higher than 16 which were selected as final set of indicators. The list was organized using scores ranking in ascending order within each model composition. Kendall’s concordance coefficient was 0.218 for all indicators. For all model composition, Kendall’s concordance coefficients were statistically significant.

**Table 4:** The final set of indicators and results of Kendall’s concordance coefficient

Model position	Indicator	Overall score (Mean ± SD)	Kendall’s concordance coefficient
Driving force	Times of enforcement supervision of tobacco control	17.3 ± 4.4	0.171^*^
	Posting rate of no-smoking signs in public places	17.1 ± 3.1	
	Number of complaints through complaint hotline	16.3 ± 4.1	
Public service	Number of smoking cessation clinics	17.2 ± 2.7	0.155^*^
	Person-time of cessation services by smoking cessation clinics	16.4 ± 3.1	
	Percentage of residents who ever have seen or listen to tobacco control information through media	16.1 ± 2.7	
Pressure	Cigarette consumption	18.5 ± 2.4	0.261^***^
	Cigarette price	17.1 ± 3.0	
	Cigarette price elastic coefficient	16.6 ± 2.6	
	Cigarette tax	16.5 ± 3.8	
Awareness and behavior	Smoking prevalence	19.6 ± 0.8	0.345^***^
	Awareness rate of the hazards of smoking among residents	17.9 ± 2.3	
	Cessation rate	17.0 ± 3.3	
	Planning cessation rate	16.0 ± 3.2	
Status	Indoor pollutant level	16.7 ± 3.6	0.240^**^
	“Three without” indicator	16.0 ± 3.3	
Exposure	Second-hand smoke exposure rate	17.2 ± 2.5	0.169^*^
	Content of cotinine in non-smokers’ saliva	16.4 ± 3.9	
Effect	Mortality rate of smoking related diseases	19.5 ± 1.2	0.338^***^
	Hospital admission rate of smoking related diseases	18.8 ± 2.2	
	Hospital admission fees of smoking related diseases	18.4 ± 2.7	
	Incidence of smoking related diseases	16.6 ± 2.3	
	Premature death risk of smoking related diseases	16.1 ± 2.1	
Overall	-	-	0.218^***^

***denotes *P* < 0.001, ** denotes *P* < 0.01, *denotes *P* < 0.05

#### Driving force

Times of enforcement supervision of tobacco control in Beijing reflects the intensity of implementation of the smoking ban in public places. During June 2015 to May 2020, data could be obtained from the routine surveillance system of the health supervising department while from June 2020 onwards, data collection might require more efforts as the regulatory power had been shifted to street level administrative units. The tobacco control complaint hotline indicates both the number of violations of the smoking ban and the extent of public participation of tobacco control. Data could be accessed through the hotline administrative department. Posting rates of no-smoking signs in public places presents the implementation extent of the smoking ban. Data could be obtained from monitoring data from the tobacco control department.

#### Public service

Number of smoking cessation clinics indicates the supply level of cessation services in Beijing and person-time of receiving cessation intervention in medical institutions indicates the service utilization. Both could be obtained from cessation service administrative department. Percentage of residents who ever have seen or listen to tobacco control information through media reflect their access to tobacco control publicity services. Data could be accessed through Beijing’s Adults Tobacco Survey.

#### Pressure

Cigarette consumption, cigarette price and cigarette tax can be obtained from the Tobacco Yearbook. Cigarette price elastic coefficient indicates change in percentage of cigarette sales as a result of a change in price. The coefficient could be calculated using the conventional demand model or the addictive demand model.

#### Awareness and behavior

Smoking prevalence can be calculated as the number of current smokers divided by participants surveyed. Awareness rate of the hazards of smoking among residents is the percentage of participants surveyed whom correctly answered that smoking could lead to the following diseases: lung cancer, acute myocardial infarction, stroke and erectile dysfunction. Cessation rate is calculated as the number of people successfully quit smoking divided by the number of ever smokers. Planning cessation rate is the percentage of smokers planning to quit among current smokers. The data source of all indicators within this model composition was Beijing’s Adult Tobacco Survey.

#### State

Indoor pollutant level reflects the status of smoking pollutant in indoor places. This could be obtained from specific surveys undertaken by the tobacco control department. The “Three without” indicator shows the percentage of public places without smell of cigarette, butt or smoking set. It indicates both the pollutant level and the implementation level of the smoking ban. Data can be accessed through investigation data from the tobacco control department.

#### Exposure

Second-hand smoke exposure rate is the percentage of nonsmokers who are exposed to second-hand smoke in the following public places: indoor workplaces, government buildings, medical institutions, restaurants, entertainment places and schools. This can be obtained through the Beijing’s Adult Tobacco Survey. Content of cotinine in non-smokers’ saliva measures the cotinine level in non-smoking restaurant service staffs’ saliva, indicating their exposure to second-hand smoke. Data could be accessed through a specific survey by the health department.

#### Effect

Mortality rate of smoking related diseases could be obtained from Beijing’s death record system. With information of death age and population in all age groups, premature death risk can be calculated. For incidence and hospital admission rate of smoking related diseases, rates of cardiovascular diseases could be accessed through Beijing’s chronic diseases surveillance system and cancer rates can be obtained from Beijing’s cancer registration system. Hospital admission fees of smoking can be obtained through Beijing’s medical record system.

## Discussion

This study developed a set of indicators for the scoping of HIA of a comprehensive tobacco control policy in Beijing. The final set of indicators reflecting different aspects of HIA were adequately defined, important, accurate and stable. The selected indicators were highly reliable due to the high level of concordance and the recognized authority of 13 experts with a multidisciplinary background.

The integrated tobacco control health impact framework represents a reliable framework to integrate the information gathered (Fig. 2). It recognizes that the link between tobacco control policy and health effect are mediated by many different factors operating through a chain of public service, status, awareness and behavior and exposure. The DPSEEA model was developed from the effect of classical environmental exposures on health, i.e., air pollution and respiratory diseases. The application of this model to tobacco control policy is new and its combination with the DOH needs a sustained effort to conceptualize the cause-effect chain of behavior, environment and public services and health effects. As the scope of the indicators was to monitor the changes introduced by the comprehensive tobacco control policy more than to measure the presence of the action itself, the working group decided not to propose the action indicators. While for the DOH, since the structural environment determinant is overlap with the status component in the DPSEEA model and there is no indicator found matching the mutual support determinant, the working group decided not to propose them. It is noteworthy that although we did not include individual characteristics in the final framework, we suggested the potential for the awareness and behavior and the effect indicators to be stratified with age, sex and education level to measure the equity of different subgroups on the beneficial of tobacco control policy. This is of great importance to promote equity in resources allocation, service utilization and health outcomes when making and implementing tobacco control policies. The current study shared some similarities with Costa et al. [21] HIA of Portuguese tobacco control legislation in effect indicators, tobacco consumption, and behavior indicators but this study is more comprehensive by considering driving force indicators, other pressure indicators, public service indicators, status indicators and exposure indicators. In addition, our indicators cover three out of the four WHO recommendation essential indicators [22] excluding the tobacco control policies as the current study’s focus is a specific tobacco control policy. All three indicators ranked high among our results which indicated the consistency in opinion towards indicators selection for tobacco control evaluation between experts in our study and the WHO. Smoking prevalence ranked first, mortality rate ranked the second and tobacco consumption ranked the fourth.

### Strengths and limitations

In the study, a working group with a diverse background was used, and a high response rate of 100% was attained. The research was organized and transparent, which gave the findings more credibility. The high levels of engagement seen in this study suggest the possibility of creating a common understanding of what is necessary to assess tobacco control policy as well as developing crucial indicators for policies aimed at enhancing health. The resulting collection of indicators has significant Kendall’s concordance coefficients and is valid. To the best of our knowledge, this study is the first to suggest a group of indicators that are supported by research and may be used in an HIA to assess tobacco control programs. The group of indicators can be used to assess the effects of tobacco control policies on health across all seven dimensions and along the entire causal chain from the policies to the consequences on health. The extensive collection of indicators lays the stage for a more thorough assessment of tobacco control policy in a major city.

However, because some of the variables tend to reflect what the working group considered pertinent regarding the Beijing’s setting, the external validity of the study’s conclusions may be constrained. Depending on the expertise and the circumstances, it’s conceivable that some indicators would be viewed as important. This study should be utilized as a springboard for discussions about the health effect indicator sets that Beijing’s statistics systems would use, adapt to local contexts, involve local panels of stakeholders, and highlight research objectives related to tobacco control. Due to the lack of a pertinent indicator, another restriction is that we did not incorporate the mutual support determinant in the composition of our framework. Future study may gather additional data on this issue and establish pertinent indicators.

## Conclusion

This study identified a set of twenty-three indicators to be used in the scoping of HIA of a comprehensive tobacco control policy in Beijing based on a tobacco control health impact conceptual framework. The set of indicators achieved high scores and statistically significant consistency, and it has great potential to promote the evaluation of tobacco control policy in a global city. By monitoring and evaluation, the implementation of the comprehensive tobacco control policy might perform better. And as more evidence on the health benefits of the comprehensive tobacco control policy is added, more cities or countries might adopt a comprehensive tobacco control policy to protect people from tobacco. Further study might use the set of indicators for HIA on tobacco control policy to analyze empirical data.

## Data Availability

The dataset supporting the conclusions of this article is included within the article.
